# Polysaccharide, Conjugate, and mRNA-based Vaccines are Immunogenic in Patients with Netherton Syndrome

**DOI:** 10.1007/s10875-024-01828-0

**Published:** 2024-10-30

**Authors:** Anouk E. M. Nouwen, Luca M. Zaeck, Renske Schappin, Daryl Geers, Lennert Gommers, Susanne Bogers, Willem A. Dik, Suzanne G. M. A. Pasmans, Corine H. GeurtsvanKessel, Rory D. de Vries, Virgil A. S. H. Dalm

**Affiliations:** 1 Department of Dermatology, Erasmus University Medical Center, Rotterdam, The Netherlands; 2 Department of Viroscience, Erasmus University Medical Center, Rotterdam, The Netherlands; 3 Laboratory Medical Immunology, Department of Immunology, Erasmus University Medical Center, Rotterdam, The Netherlands; 4 Department of Dermatology-Center of Pediatric Dermatology/Center of Rare Skin Diseases, Erasmus University Medical Center-Sophia Children’s Hospital, Rotterdam, The Netherlands; 5 Department of Immunology, Erasmus University Medical Center, Dr. Molewaterplein 40, Rotterdam, 3015 GD The Netherlands; 6 Department of Internal Medicine, Division of Allergy & Clinical Immunology, Erasmus University Medical Center, Rotterdam, The Netherlands

**Keywords:** Immunodeficiency, Netherton syndrome, COVID-19, *SPINK5*, Vaccination response

## Abstract

**Background:**

Netherton syndrome (NS) is a rare, severe genetic skin disorder, currently classified as an inborn error of immunity (IEI) due to previously reported immune dysregulation. We recently reported the results of an immunological evaluation showing no evidence for a relevant B- and/or T-cell mediated immunodeficiency, but immune responses after vaccination were not evaluated in that study. Therefore, we evaluated immune responses to three vaccine platforms in adult NS patients to further investigate the presence of a clinically relevant B- and/or T-cell immunodeficiency.

**Methods:**

Vaccination responses in eight adult NS patients were assessed in a cross-sectional study performed between January and August 2022. Clinical patient data were retrospectively retrieved from electronic patient files. Immune responses to a polysaccharide *Streptococcus pneumoniae* vaccine (PPV23) and conjugate *Haemophilus influenzae* type b vaccine (ActHiB) were measured. SARS-CoV-2-specific (functional) antibody and T-cell responses following booster vaccination with an mRNA-based COVID-19 vaccine were compared to controls.

**Results:**

None of the included patients suffered from recurrent and/or severe infections that could be attributed to a B- and/or T-cell immunodeficiency. ActHiB induced immune responses were normal in 7/7 NS patients. PPV23 induced responses were absent in 1/7, diminished in 2/7, and normal in 4/7 patients. Levels of SARS-CoV-2-specific binding and neutralizing antibodies after mRNA-based COVID-19 booster vaccination in NS patients were comparable to controls. SARS-CoV-2-specific CD4 + T-cell responses were detectable in all NS patients. In contrast, SARS-CoV-2-specific CD8 + T-cell responses were detectable in only 2/6 NS patients. T-cell responses to a positive control antigen pool were comparable to controls.

**Conclusions:**

Vaccine-induced immune responses were detectable after polysaccharide, conjugate and mRNA-based vaccination in our cohort of NS patients. A spectrum of responsiveness to vaccine challenges was found, with the ranges of vaccine responses overlapping those demonstrated in healthy control populations.

**Supplementary Information:**

The online version contains supplementary material available at 10.1007/s10875-024-01828-0.

## Introduction

Netherton syndrome (NS; OMIM#256500) is a rare, severe genetic skin disorder with an estimated prevalence of 1–9/1.000.000 [1]. In total, 21 patients in the Netherlands are currently diagnosed with NS [2]. NS is caused by pathogenic variants in the Kazal type 5 (*SPINK5*) gene on chromosome 5q32, which encodes the serine protease inhibitor lymphoepithelial Kazal-type–related inhibitor (LEKTI) [3–5]. Functional loss of LEKTI results in an uninhibited activity of serine proteases, including kallikreins such as kallikrein-related peptidase 5 [6–8]. As a consequence, the skin barrier is severely impaired [5, 9]. Secondary secretion of pro-allergic and pro-inflammatory cytokines, such as thymic stromal lymphopoietin, tumor necrosis factor-a, intercellular adhesion molecule 1, and interleukin-8, leads to inflammatory and allergic predisposition [5, 10, 11]. NS is clinically characterized by congenital ichthyosiform erythroderma, trichorrhexis invaginata, and atopic manifestations with eosinophilia and high IgE levels [12, 13]. However, clinical manifestations may be highly heterogeneous between individuals [14], disease manifestation may evolve over time, and patients with less severe disease may survive into adulthood, while patients with severe disease may die at young age.

NS has been classified as an inborn error of immunity (IEI) due to abnormalities in numbers of immune cells in peripheral blood, functionality of these immune cells and disturbed mucosal immunity [15]. Specifically, decreased numbers of switched and non-switched circulating B-cells, a reduced proportion of naïve CD4 + T-cells, and impaired NK-cell cytotoxicity have been described [16, 17]. Furthermore, several clinical features suggest a B- and/or T-cell immunodeficiency, which include recurrent skin, ear, nose and throat (ENT) infections, respiratory tract infections, and systemic infections [13, 17, 18]. However, a recent immunological evaluation of the Dutch NS patient cohort, including both adult patients and children(*N* = 14) showed that absolute numbers of lymphocyte subsets and serum immunoglobulin levels were within normal range. Instead, the study concluded that an increased risk of infections most likely resulted from skin barrier disruption [13]. Nonetheless, the antigen-specific response after vaccination was not evaluated. In previous studies, the antigen-specific response after vaccination in NS patients has been scarcely described, with conflicting results [16–20].

Here, we performed a cross-sectional evaluation of immune response to three types of vaccines in patients with NS to further explore the presence of a clinically relevant B- and/or T-cell immunodeficiency. Immune responses after vaccination with a polysaccharide *Streptococcus pneumoniae* vaccine (PPV23) and a conjugate *Haemophilus influenzae* type b vaccine (ActHiB) were evaluated. These vaccines are commonly used for assessing T-cell independent and T-cell dependent antibody responses, respectively [21]. Additionally, SARS-CoV-2-specific binding and neutralizing antibody and T-cell responses after booster vaccination with an mRNA-based COVID-19 were measured.

## Materials and Methods

### Study Population

Immune responses to vaccination were evaluated in eight adult patients with NS in a cross-sectional study performed between January 2022 and August 2022 at the Erasmus University Medical Center Rotterdam, an acknowledged national center of expertise for NS. In all patients a genetic diagnosis was confirmed. The study was approved by the Medical Ethical Committee of the Erasmus University Medical Center (MEC-2022-0616; MEC-2020-0264, MEC-2022-0462, MEC- 2013-026 and MEC-2021-0050), Rotterdam, the Netherlands, and was performed in accordance with the Declaration of Helsinki. All patients and healthcare workers controls provided written informed consent.

### Clinical Data Collection

Clinical data of NS patients was retrieved from electronic patient files, and included the following information: disease severity, medication use, medical history, comorbidities, and results of blood examinations performed within clinical care. Disease severity was retrospectively evaluated using previously collected medical photographs, which were scored according to the Ichthyosis Area and Severity Index (IASI) [22]. Intensity of erythema and scaling is scored at different pre-defined body parts, also taking into account body surface area involved. Pain and pruritus within the last 24 h were assessed by numerical rating scale (NRS). Patient report peak pruritus by scoring between 0 and 10 Clinical care blood examinations included measurement of serum immunoglobulin levels (i.e., IgE, IgM, IgA, IgG), and diagnostic vaccination responses to PPV23 and ActHiB. Medical history included the COVID-19 vaccination history, patients’ self-reported SARS-CoV-2 infections, and experienced symptoms during SARS-CoV2- infection. Warning signs for primary immunodeficiency disease (PID) in adults were evaluated according to the European Society for Immunodeficiencies (ESID) criteria [23].

### Vaccination Response to PPV23 and ActHiB

Immune responses to a polysaccharide vaccine against *Streptococcus pneumoniae* (Pneumovax 23; PPV23) and a conjugate vaccine against *Haemophilus influenza* type b (ActHiB) were measured and evaluated as part of standard clinical work-up for suspicion of immunodeficiency. Patients were vaccinated with PPV23 and ActHiB and after 4 to 6 weeks, antigen-specific antibody concentrations against *S. pneumoniae* and *H. Influenza* type b were measured and compared to pre-vaccination concentrations. Using a Luminex assay, an increase in antibody concentration of at least 2-fold, reaching at least 1.00 µg/mL for a minimum of 9 out of 16 measured *S. pneumoniae* serotypes (1, 3, 4, 5, 6B, 7 F, 8, 9 V, 14, 15B, 18 C, 19 A, 19 F, 20, 23 F, 33 F) was determined as a normal response to vaccination according to the in-house ISO 15,189 accredited protocol [24, 25]. This protocol was adapted from Borgers et al. [26]. Antigen-specific antibody concentrations against *H. Influenza* type b were measured by ELISA. Antibody concentrations reaching at least 1.00 µg/mL were classified as a normal response to ActHiB vaccination [27].

### Vaccination Response to mRNA-based COVID-19 Vaccination

All participants received an mRNA-based primary vaccination regimen against COVID-19 (mRNA-1273 or BNT162b2) via the Dutch vaccination program between March 2021 and June 2021. Participants received an additional COVID-19 mRNA-based (mRNA-1273 or BNT162b2) booster vaccination between December 2021 and February 2022. Serum and peripheral blood mononuclear cells (PBMC) were collected 4 to 8 weeks after booster vaccination. PBMC were stored in liquid nitrogen until further use.

### Humoral Immune Response to mRNA-based COVID-19 Vaccination

Spike (S)-specific binding antibodies were measured by Liaison SARS-CoV-2 TrimericS immunoglobulin G (IgG) assay (DiaSorin) as previously described [28]. The lower limit of detection (LLoD) and the responder cutoff were defined as 4.81 BAU/ml and 33.8 BAU/ml, respectively. The presence of neutralizing antibodies against the ancestral SARS-CoV-2 (D614G) was measured in a plaque reduction neutralization test (PRNT) as previously described [28, 29]. Briefly, ancestral SARS-CoV-2 was cultured from clinical material and sequence was confirmed (GISAID: hCov-19/Netherlands/ZH-EMC-2498). The human airway Calu-3 cell line (ATCC HTB-55) was used to grow virus stocks and for PRNT. Calu-3 cells were cultured in OptiMEM supplemented with GlutaMAX (Gibco), penicillin (100 units/mL, Capricorn Scientific), streptomycin (0.1 mg/mL, Capricorn Scientific), and 10% fetal bovine serum (FBS; Sigma). Two-fold dilution series of heat-inactivated sera were prepared in OptiMEM without FBS. The dilution range of a sample was dependent on its S-specific binding antibody titers (< 1500 BAU/mL: 1:10–1:1280; 1500–6000 BAU/mL: 1:80–10240; >6000 BAU/mL: 1:640–1:81920). Following the addition of 400 plaque-forming units (PFU) in an equal volume of OptiMEM, the virus-serum mix was incubated for 1 h at 37 °C before transfer of 100 µL to confluent Calu-3 and incubation for 8 h at 37 °C. Next, the cells were fixed with 10% neutral-buffered formalin for 30 min, permeabilized in 70% ethanol, and plaques were stained with a polyclonal rabbit anti-SARS-CoV-2 nucleocapsid antibody (Sino Biological) and a peroxidase-conjugated goat anti-rabbit secondary antibody (Dako). The signals were developed using a TMB substrate (TrueBlue; SeraCare/KPL) and the number of plaques was quantified using an ImmunoSpot Image Analyzer (CTL Europe GmbH). The dilution at which a 50% reduction of infected plaques (PRNT50) was reached compared to the infection control was estimated by determining the proportionate distance between two dilutions from which an endpoint titer was calculated. When no neutralization was observed, the PRNT50 was given a value of 10. Humoral immune responses from Netherton patients were compared to a control cohort of 18 healthcare workers (HCW), which was matched for age, sex, and infection status (age range: 24–51 years; 12 men and 6 women) The HCW study was approved by the institutional review board of the Erasmus MC (medical ethical committee, MEC-2020-0264).

### T-cell Response to mRNA-based COVID-19 Vaccination

SARS-CoV-2-specific T-cell responses were assessed in PBMC collected 4–8 weeks after booster as described previously [30]. Briefly, 1 × 10^6^ PBMC were stimulated in 200µL RPMI1640 medium supplemented with Glutamax (Gibco), penicillin (100 units/mL, Capricorn Scientific), streptomycin (0.1 mg/mL, Capricorn Scientific), and 10% human serum (R10H) with an overlapping peptide pool (15-mers) covering the S protein of the ancestral, Omicron BA.1, or Omicron BA.5 variants. A CEFX super stimulation pool containing immunogenic peptides from common human pathogens and commensals was included as a positive control. Cells were stimulated with an equimolar concentration of DMSO as negative control. PBMC were incubated at 37℃ for 20 h prior to staining for phenotypic lymphocyte and activation markers. Cells were stained following ex vivo stimulation at 4 °C for 30 min with the following antibodies: anti-CD3PerCP (Clone SK7, BD, 1:25), anti-CD4V450 (Clone L200, BD, 1:50), anti-CD8FITC (Clone DK25, Dako, 1:25), anti-CD45RAPE-Cy7 (Clone L48, BD, 1:50), anti-CCR7BV711 (Clone 150503, BD, 1:25), anti-CD69APC-H7 (Clone FN50, BD, 1:50), anti-CD137PE (Clone 4B4-1, Miltenyi, 1:50), and anti-OX40BV605 (Clone L106, BD, 1:25). LIVE/DEAD™ Fixable Far Red Cell staining was included (APC, Invitrogen, 1:1000) for exclusion of dead cells. T-cells were gated as LIVE CD3 + cells and subdivided into CD4 + or CD8 + subsets. Within the CD4 + and CD8 + sub-population, memory cells were gated by the exclusion of CCR7 + CD45RA + naïve T-cells. Within the memory population, SARS-CoV-2-specific T-cells were identified as activation induced marker (AIM) positive as CD137 + OX40 + for CD4 + or CD137 + CD69 + for CD8 + T-cells. On average 175,000 cells were acquired on a FACSLyric (BD) after data clean-up in the time gate. NS patient 2, was excluded from further analysis because of low cell counts. AIM + T-cells percentages from NS patients were compared to an age-matched cohort of 16 healthcare workers (MEC-2022-0462; age-matched; age range: 29–59; 4 men and 12 women).response. AIM expression was analyzed with FlowJo software version 10.8.1; the gating strategy is depicted in Figure S1. Data is presented as the percentage of double positive (AIM+) CD4 and CD8 T-cells.

### Analysis

Characteristics of NS patients, and results of the vaccination responses to PPV23 and ActHiB were analyzed descriptively. Median number of days between COVID-19 mRNA-based booster vaccination and antibody response measurement are presented as median (interquartile range (IQR)). Levels of SARS-CoV-2 S-specific binding and neutralizing antibodies, and AIM + T-cells percentages are reported as geometric mean titer (GMT) or geometric means (GM), respectively, with 95% CI, and compared between NS patients and the control group by Mann-Whitney U test. P-values less than 0.05 were considered statistically significant. Statistical analyses were performed and graphs were prepared with Graphpad PRISM version 10 (San Diego, CA, USA).

## Results

### Clinical Patient Characteristics

Eight adult NS patients (age range: 27–52 years; 5 men and 3 women) were included (Table 1). Disease severity, pruritus and pain varied between patients. One patient was treated with a biological (Ixekizumab; anti-IL17) at the time of vaccination response measurements. No other systemic immunosuppressants were used by NS patients within at least two weeks and/or 5 half-lives prior to the measurements. No patient scored positive for any of the ESID warning signs for adult PID [23] or recurrent gastro-intestinal tract infections. One patient (patient 2) had a history of recurrent cutaneous malignancies and was diagnosed with a metastasized cutaneous squamous cell carcinoma shortly after vaccination response evaluation. None showed a distinct antibody deficiency. All patients had normal to high serum levels of IgG and IgA. Two NS patients (patients 5 and 6) had reduced IgM serum levels (Table 2).

**Table 1:** Patient characteristics

Patient	Sex	Age (y)	Age (Y)	IASI	NRS pruritus^a^ (0–10)	NRS pain^a^ (0–10)	Current medication use	Total IgE (kU/L)	Warning signs for PID in adults^b^						Q. Gastro-intestinal infection
			at diagnosis						Q1	Q2	Q3	Q4	Q5	Q6	
1	F	52	40	19.4	8	4	Topical corticosteroids, emollients	127	No	No	No	No	No	No	No
2	M	48	21	30.7	3–4	0	Acitretin 10 mg daily, emollients	51	No	No	No	No	No	No	No
3	M	27	0,5	15.2	6	0	Ixekixumab 80 mg once every 5 weeks, emollients, antihistamines	> 25,000	No	No	No	No	No	No	No
4	M	41	1	4.6	4	2	Topical corticosteroids, emollients	3034	No	No	No	No	No	No	No
5	M	45	23	11.7	7–8	6	Topical emollients, antihistamines, antibiotic (doxycycline), topical coal tar cream, coal tar shampoo	15,417	No	No	No	No	No	No	No
6	M	29	3	16.4	2.5-3	1-1.5	Topical corticosteroids, emollients, topical coal tar	> 25,000	No	No	No	No	No	No	No
7	F	30	1	23.6	6	4–5	Topical corticosteroids, emollients, topical tacrolimus, fucidin cream, antibiotic (doxycycline), anthistamines	10,291	No	No	No	No	No	No	No
8	F	28	0	31.9	8	5	Topical corticosteroids, emollients, topical zincoxide	21,765	No	No	No	No	No	No	No

a = within the last 24 h. b = The 6 ESID warning signs for adult primary immunodeficiency diseases [23]. Q1: Four or more infections requiring antibiotics within one year (otitis, bronchitis, sinusitis, pneumonia), Q2: Recurring infections or infection requiring prolonged antibiotic therapy, Q3: Two or more severe bacterial infections (osteomyelitis, meningitis, septicemia, cellulitis), Q4: Two or more radiologically proven pneumonia within 3 years, Q5: Infection with unusual localization or unusual pathogen, Q6: Primary immunodeficiency disease in the family. Question on gastro-intestinal infections: Gastro-intestinal infection caused by Giardia lamblia, Campylobacter, Shigella or Yersinia, or other pathogens. Abbreviations: F, female; M, male; y, years; IASI, Ichthyosis Area and Severity Index; NRS, numerical rating scale; IgE, immunoglobulin E; PID, Primary immunodeficiency disease; Q, Question

**Table 2:** Immunoglobulin levels and vaccination responses to PPV23 and ActHiB in patients with Netherton syndrome

Patient	Serum immunoglobulin levels^1^ (g/L)			Polysaccharide vaccine	Conjugate vaccine
	IgM (0.45–2.30)^2^	IgG (7.0–16.0)^2^	IgA (0.76–3.91)^2^	PPV23	ActHiB
1	1.76	10.8	3.33	Inadequate	Adequate
2	0.58	*16.5*	*6.65*	Diminished	Adequate
3	1.15	10.3	3.34	Adequate	Adequate
4	0.48	8.8	2.09	Adequate	Adequate
5	**0.22**	14.8	1.78	n.d.	n.d.
6	**0.29**	14	2.48	Diminished	Adequate
7	1.03	7.3	1.16	Adequate	Adequate
8	1.51	9.9	2.87	Adequate	Adequate

^1^ =Total IgM, IgG, and IgA serum levels were measured by using immunonephelometry with a Siemens BN II nephelometer according to manufacturer’s instructions. ^2^ = Normal values for adults. Values below the normal ranges are depicted in bold; values above the normal range are depicted in italics. Abbreviations: IgM, immunoglobulin M; IgG, immunoglobulin G; IgA, immunoglobulin A; PPV23 = pneumococcal polysaccharide vaccine 23; ActHiB = Haemophilus b conjugate vaccine; n.d., not determined

### Vaccination Response to PPV23 and ActHiB

**Fig. 1:**
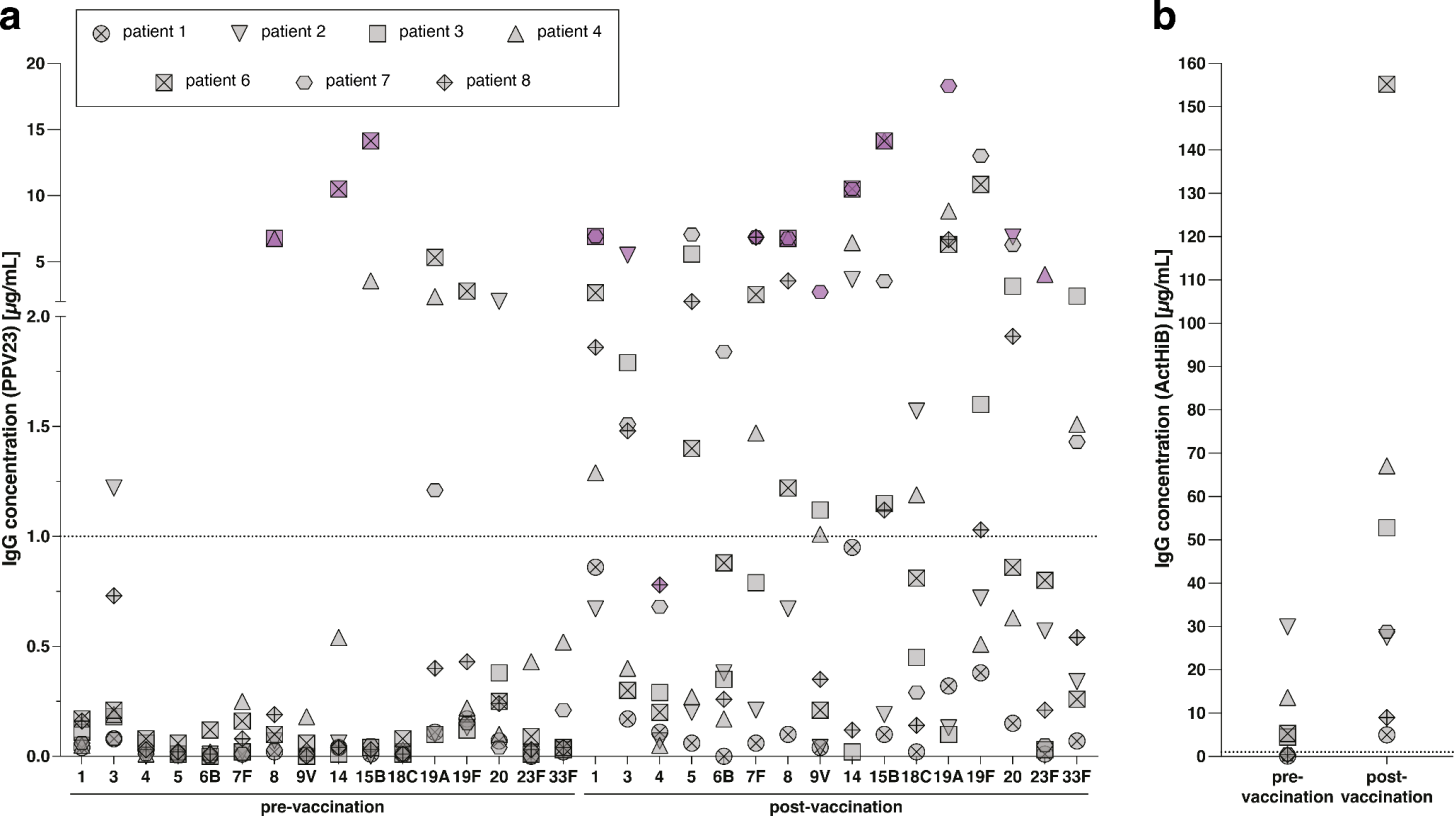
Vaccination responses to PPV23 and ActHiB. (**a**) Comparison of antibody concentration (µg/mL) per pneumococcal serotype before and after PPV23 vaccination. Samples exceeding the serotype specific upper limit of detection are marked by a purple symbol. (**b**) Comparison of antibody concentration (µg/mL) before and after ActHiB vaccination. Symbols show individual data points. Each Netherton syndrome (NS) patient has their own unique symbol as indicated by the legend. Patients 3 and 8 received prior *Haemophilus influenza* type b vaccination, which was part of the Dutch National Immunisation Programme (DNIP) during their childhood. Patient 5 did not receive PPV23 or ActHiB vaccination, antibody concentrations were thus not determined. The dotted line represents 1.00 µg/mL. PPV23 = Pneumovax 23, polysaccharide vaccine against *Streptococcus pneumoniae*; ActHiB = conjugate vaccine against *Haemophilus influenza* type b. Individual patient kinetics pre- and post-vaccination are shown in Supplementary Figure S2

Two patients (patient 3, 8) were vaccinated according to the Dutch National Immunisation Programme (DNIP) during their childhood at the time that *Haemophilus influenza* type b vaccination was part of the DNIP. None reported to have received additional prior vaccination against *Haemophilus influenza* type b or *Streptococcus pneumoniae*. In 7 NS patients, responses to PPV23 and ActHiB were evaluated (Table 2; Fig. 1, and Table S1 and Figure S2). Four out of 7 patients (patient 3, 4, 7, and 8) showed normal responses to PPV23. Normal response to PPV23 is described and defined in the Methods section. In patient 1, responses to PPV23 were absent. Although the fold-increase in antibody concentration was at least 2 for most serotypes in this patient, concentrations did not reach IgG levels of 1.00 µg/mL or higher in any of the tested serotypes. Patient 2 and 6 showed diminished responses. Patient 2 reached IgG levels of 1.00 µg/mL or higher for 2 serotypes, which were below 1.00 µg/mL before vaccination, but fold-increases in antibody concentrations were at least 4 for most serotypes. Patient 6 reached IgG levels of 1.00 µg/mL or higher for 4 serotypes, which were below 1.00 µg/mL before vaccination. All NS patients reached protective antibody concentrations against *H. influenza type b* (i.e., at least 1.00 µg/mL). In 4 out of 7 patients the antibody concentration against *H. influenza type b* was already higher than 1.00 µg/mL pre-vaccination. The fold-increase in antibody concentration in patients 3, 4, and 6 was at least 4.9 (Table S1). In patient 2, antibody concentration post-vaccination was slightly lower than pre-vaccination.

### COVID-19 Baseline Characteristics

**Fig. 2:**
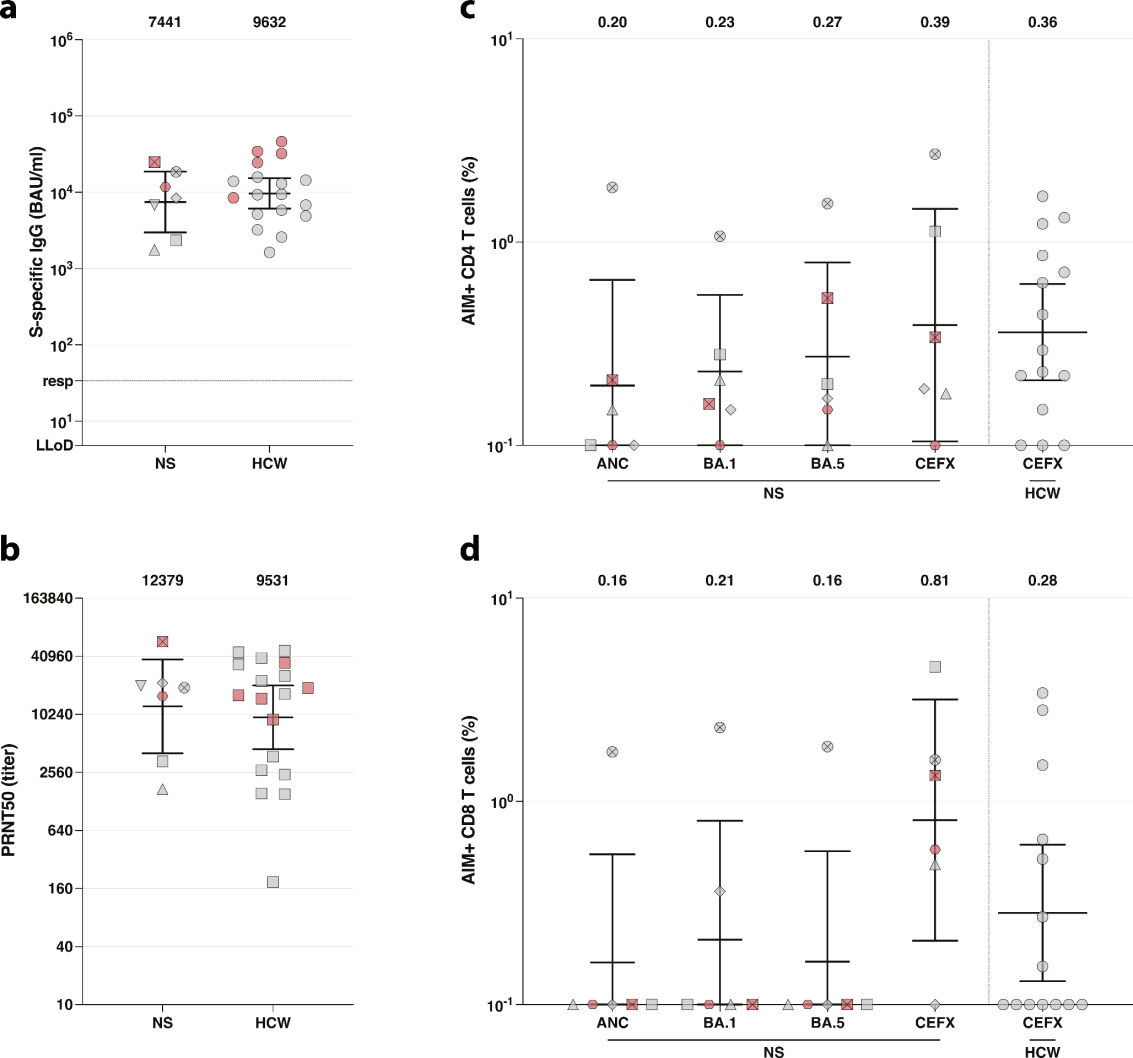
SARS-CoV-2–specific immune responses after booster vaccination. (**a**) Comparison of S-specific IgG antibodies (GMT ± 95% CI) after COVID-19 booster vaccination between NS patients and HCW. LLoD is 4.81 BAU/ml, responder (resp) cut-off is 33.8 BAU/ml (dotted line). (**b**) Comparison of neutralizing antibodies (GMT ± 95% CI) determined by PRNT after COVID-19 booster vaccination between NS patients and HCW. When no neutralization was observed, PRNT50 was given a value of 10. (**c**, **d**) Percentage of AIM + T-cells against ancestral SARS-CoV-2, and Omicron sub-lineages BA.1 and BA.5 after COVID-19 booster vaccination. A CEFX peptide pool was included as positive control. Data (GM ± 95% CI) is presented as the percentage of AIM + CD4 (**c**) and CD8 T-cells (**d**). LLoD is set at 0.1%. Symbols show individual data points. Individuals with a history of SARS-CoV-2 infection were marked by a red symbol. Each NS patient was given a unique symbol. Bold numbers above the plots represent the respective geometric mean (titer). GMT = geometric mean titer; GM = geometric mean; LLoD = lower limit of detection; S = Spike; BAU = binding arbitrary units; HCW = healthcare workers; PRNT = plaque reduction neutralization test, AIM = activation-induced markers; ANC = ancestral SARS-CoV-2 strain

In 7 NS patients SARS-CoV-2-specific immune responses were measured 4–8 weeks after receiving an mRNA-based booster vaccination (Table 3; Fig. 2). Patient 8 did not receive a booster vaccination, but S-specific IgG antibodies were evaluated as part of clinical follow-up. Three patients reported a SARS-CoV-2 infection before booster vaccination, of which one infection occurred within three months before measurement and another one with patient 8, who did not receive a booster vaccination. Reported symptoms during COVID-19 included fever, fatigue, a sore throat, congestion, cough, loss of taste and smell, headache, and exacerbation of the skin disease. In all cases, the symptoms lasted for a maximum of two weeks. No patients were admitted to the hospital/ICU or required additional treatments for the SARS-CoV-2 infection. A comparable ratio of individuals who received a booster vaccination and had a history of SARS-CoV-2 infection (5/18 [27.8%] versus 2/7 [28.6%] in the NS cohort) were included in the matched control group for humoral immune responses.

**Table 3:** COVID-19 characteristics of Netherton syndrome patients

Patient	Primary COVID-19 vaccination regimen (*n*)	COVID-19 booster vaccination (*n*)	Days between booster and sample collection	Reported SARS-CoV-2 infections, < 3 months	Reported SARS-CoV-2 infection since start of the pandemic (*n*)	SARS-CoV-2 S-specific IgG antibodies (BAU/mL)	Neutralizing antibodies against ancestral SARS-CoV-2 (titer)
1	BNT162b2 (2)	mRNA-1273 (1)	32	No	No	18,400	19,356
2	BNT162b2 (2)	BNT162b2 (1)	42	No	No	6780	20,198
3	BNT162b2 (2)	BNT162b2 (1)	49	No	No	2350	3330
4	BNT162b2 (2)	BNT162b2 (1)	29	No	No	1770	1722
5	BNT162b2 (2)	mRNA-1273 (1)	32	No	No	8390	21,624
6	BNT162b2 (2)	BNT162b2 (1)	43	Yes	Yes (1)	24,800	58,005
7	BNT162b2 (2)	BNT162b2 (1)	36	No	Yes (1)	11,700	15,842
8^a^	mRNA-1273 (2)	NA	NA	No	Yes (3)	1860	n.d.

Abbreviations: n = absolute number; IgG, immunoglobulin G; BAU, binding arbitrary units; NA, not applicable; n.d., not determined. a = This patient did not receive a SARS-CoV-2 mRNA booster vaccination. SARS-CoV-2 S-specific antibodies were evaluated as part of clinical follow-up

### SARS-CoV-2-specific Antibody Responses after Booster Vaccination

SARS-CoV-2 S-specific binding and neutralizing antibodies were measured in sera collected from 7 NS patients and 18 matched controls (Table 3; Fig. 2a-b). The median number of days between COVID-19 mRNA-based booster vaccination and antibody response measurement was 36 days (IQR 32–43) for NS patients and 40 days (IQR 37.5–41) for matched controls, respectively. The GMT of SARS-CoV-2 S-specific binding IgG antibodies of controls was 9,632 BAU/mL (95% CI, 6,100 − 15,209); NS patients had a similar GMT (7,441 BAU/mL; 95% CI, 2,977 − 18,600; *P* = 0.61). Neutralizing antibody levels against ancestral SARS-CoV-2 were also comparable (*P* = 0.84) between NS patients (GMT = 12,379; 95% CI, 4,036–37,970) and matched controls (GMT = 9,531; 95% CI, 4,458 − 20,380).

### SARS-CoV-2-specific T-cell Responses after Booster Vaccination

In 6 NS patients, virus-specific T-cell responses were measured in PBMC by an AIM assay (Fig. 2c-d). CD4 + T-cell responses after stimulation with a CEFX peptide pool, a superantigen positive control pool for T-cell stimulation, were detectable in 5/6 NS patients (GM = 0.39; 95% CI, 0.10–1.5). Comparable results were found in controls (12/15 controls [GM = 0.36; 95% CI, 0.21–0.62, *P* = 0.98, Fig. 2c]), which were independent from the control group used for the comparison of antibody responses. Virus-specific CD4 + T-cell responses against ancestral SARS-CoV-2 (GM = 0.20; 95% CI, 0.060–0.65) as well as Omicron sub-lineages BA.1 (GM = 0.23; 95% CI, 0.097–0.55) and BA.5 (GM = 0.27; 95% CI, 0.094–0.79) were detected in 3/6, 5/6, and 5/6 NS patients, respectively. In all NS patients, virus-specific CD4 + T-cell responses were measured against at least one SARS-CoV-2 variant. CD8 + T-cell responses after stimulation with a CEFX peptide pool were detectable in 7/14 controls (GM = 0.28; 95% CI, 0.13–0.61). Although CD8 + T-cell responses after stimulation with a CEFX peptide pool were detected in 5/6 NS patients (GM = 0.81; 95% CI, 0.21–3.2, *P* = 0.14 compared to controls, Fig. 2d), virus-specific CD8 + T-cell responses against ancestral SARS-CoV-2 (GM = 0.16; 95% CI, 0.047–0.55) and the Omicron sub-lineages BA.1 (GM = 0.21; 95% CI, 0.054-0.80) and BA.5 (GM = 0.16; 95% CI, 0.047–0.57) were only found in 1/6, 2/6, and 1/6 NS patients, respectively.

## Discussion

We evaluated immune responses to three types of vaccination in eight adult NS patients. Vaccination responses to PPV23 and ActHiB were normal in 4 out of 7 and 7 out of 7 patients, respectively. Levels of SARS-CoV-2-specific binding and neutralizing antibodies after COVID-19 booster vaccination were comparable to levels in controls. SARS-CoV-2-specific CD4 + T-cell responses were detectable in all NS patients; CD8 + T-cell responses were detectable in only 2/6 NS patients. Overall CD4 + and CD8 + T-cell responses after stimulation with a superantigen (CEFX) were comparable to controls. Based on these results and our previous cohort evaluation [13], we conclude as follows. Our findings of vaccination responses comparable with those found in control populations as well as the absence of clinically relevant infections that could be attributed to B- and/or T-cell immunodeficiency do not support a severe B- and/or T-cell immunodeficiency in our patient cohort. The increased risk of infections in NS patients is most likely the result of a skin barrier disruption and disturbed mucosal immunity as previously suggested [13]. Nevertheless, the sample size of this study is small, taking into consideration that subtle differences might have been missed. As clinical manifestations in NS may be highly heterogeneous in specific patients, a B- or T-cell immunodeficiency could be considered and should be evaluated in case of suspicious infections.

Limited data is available regarding immune responses to vaccination in NS patients. In this study, only 1/7 patients did not respond to PPV23 vaccination. Although patient 2 and 6 did not strictly fulfill the criteria for a normal response, a response to polysaccharide antigens was detectable. Our results are in line with previous findings by Eränkö et al., who reported that pneumococcal vaccination responses were normal to most serotypes in three NS patients aged between 10 and 17 years [16], and Smith et al., who reported low but detectable pneumococcal antibodies [19]. Contrastingly, three other studies reported poor responses to Pneumovax in 7/8 patients below the age of 12 years [17, 18, 20]. These patients, in contrast to our adult population, presented with frequent infections, including ear infections, respiratory infections, gastro-intestinal infections, skin infections, and sepsis. Of note, the number of pneumococcal serotypes tested, the cutoff levels for protection, and responder criteria varied across studies, or were not mentioned; this hindered a proper comparison with our study. The absent response to PPV23 in patient 1, and diminished response in patient 2 and 6, do not directly indicate a clinically relevant B-cell immunodeficiency. Measurement of pneumococcal polysaccharide antibody response in individuals with no medical history suggestive of PID showed that 2–34 per 100 persons would be classified with specific polysaccharide antibody deficiency, depending on the interpretation method [25]. Therefore, the results of our study are likely a representation of normal responses. Although recurrent skin infections have previously been described in our cohort [13], no clinical signs of a B- and/or T-cell immunodeficiency were present in the adult NS patients in the current study and none scored positive for any ESID warning signs for PID in adults [23]. Furthermore, no NS patients showed a distinct antibody deficiency (IgE, IgM, IgG, IgA). Regarding the response to ActHiB, similar results were seen by Stryk et al., who found low-normal to normal responses to *Haemophilus influenza* in three NS children [20]. Other T-cell dependent vaccination responses that have been measured include the response to bacteriophage phiX174, tetanus and diphtheria, with contrasting results [17–20]. Only one patient (patient 3) in this study was using systemic immunomodulatory therapies (Ixekizumab; interleukin-17 A antagonist) because of the skin condition. Previous studies did not show a negative effect of Ixekizumab on immunogenicity after vaccination [31]. No negative effects were observed for our patient 3 either.

To the best of our knowledge, this is the first study describing SARS-CoV-2-specific immune responses in NS patients. Symptoms experienced during SARS-CoV-2 infection were comparable to symptoms described in the general population [32]. In line with antibody response to PPV23 and ActHib, NS patients mounted vaccination-induced antibody responses comparable to responses measured in controls. However, this is not evidence for absence of a clinically relevant B- and/or T-cell immunodeficiency per se, as most adult patients diagnosed with a B- and/or T-cell immunodeficiency were shown to mount an immune response after mRNA-1273 vaccination as well [33]. Furthermore, neutralizing antibodies measured by PRNT were comparable to controls. This is important, as neutralizing antibodies are considered predictive of immune protection from symptomatic SARS-CoV-2 infection, indicating that COVID-19 vaccination led to protective immunity in NS [34]. It could be hypothesized that in patients with NS, considering them as being immunodeficient, there is a more rapid waning of antibody titers over time after vaccination. On the other hand, in recent studies on COVID-19 vaccination in patients with IEI, including Common Variable Immune Deficiency (CVID), X-linked agammaglobulinemia (XLA) and selective antibody deficiencies, no differences in waning of antibody titers were observed between those patients and healthy control subjects, showing that durability of antibody response after vaccination in IEI was comparable to the general population [35].

SARS-CoV-2–specific CD4 + T-cell responses to all analyzed variants were detected, with marginal differences between ancestral SARS-CoV-2, and Omicron BA.1 and BA.5 variants, implying minimal escape at T-cell level, as reported previously [28, 29]. In contrast to the SARS-CoV-2–specific CD4 + T-cell responses, SARS-CoV-2–specific CD8 + T-cell responses were only measured in 2/6 patients. However, the use peptide pools consisting of 15-mer peptides, instead of 8- to 10-mers, likely resulted in detection of lower levels of CD8 + T-cells [28, 29]. Responses of both CD4 + and CD8 + T-cells after stimulation with CEFX were comparable between NS patients and controls, suggesting that antigen-specific T-cell function in NS is normal. This is in line with the findings of Eränkö et al.., who investigated the functional capacity of T-cells stimulated with anti-CD3, anti-CD28 and anti-CD49d in six NS patients [16].

Immune responses to three types of vaccination (PPV23, ActHib, and COVID-19) were evaluated in 8 adult NS patients. The results of these responses, in combination with the clinical findings of this study and previous studies, suggest that the presence of a severe and clinically relevant B- and/or T-cell immunodeficiency in adult NS patients is unlikely. Larger international studies in both adults and children are needed to further evaluate the immune responses in NS patients in a standardized manner to confirm this data.

## Strengths and Limitations

In this study, we included half of all known Dutch adult NS patients [2] with varying disease severity. Nonetheless, the sample size in the study remains relatively small, which could be a limitation as it is assumed that very small samples undermine the internal and external validity of a study [36]. On the other side, it also known that a small sample size could provide relevant results, for instance in a study set-up to detect specified defects as in our current study [37]. We therefore feel that although the sample size is small, given the rarity of NS, our findings significantly contribute to the knowledge on B- and/or T-cell immunodeficiency in these patients. Another limitation could be that only adult patients were included in the current study, and clinical phenotypes may differ between children and adult patients, as disease manifestations may change or evolve over time However, none of the known children with NS in the Netherlands had received COVID-19 vaccination at time of our study.

One patient (patient 2) presented with a malignancy shortly after the vaccination response measurements. We cannot rule out that this might have had effect on our results. As for immune responses, SARS-CoV-2-specific responses were only measured once after booster vaccination as prior samples were not available. However, based on immune responses and similarities in response between NS patients and controls, we do not expect major deviations in NS patients during the primary vaccination regimen.

## Electronic Supplementary Material

Below is the link to the electronic supplementary material.

Supplementary Material 1

## Data Availability

No datasets were generated or analysed during the current study.
