# Supplementary material for: Polysaccharide, Conjugate, and mRNA-based Vaccines are Immunogenic in Patients with Netherton Syndrome
Source: J Clin Immunol. 2024 Oct 30;45(1):36. doi: 10.1007/s10875-024-01828-0 (PMC11525285; doi:10.1007/s10875-024-01828-0)
Supplement: Supplementary file 1 — Supplementary Material 1 [file 10875_2024_1828_MOESM1_ESM.docx]

*SUPPLEMENTARY MATERIAL BELONGING TO*

**Polysaccharide, conjugate, and mRNA-based vaccines are immunogenic in patients with Netherton syndrome**

**Short title:** Vaccination responses in Netherton syndrome

Anouk E.M. Nouwen^1^, Luca M. Zaeck^2^, Renske Schappin^1^, Daryl Geers^2^, Lennert Gommers^2^, Susanne Bogers^2^, Willem A. Dik^3^, Suzanne G.M.A. Pasmans^4^, Corine H. GeurtsvanKessel^2^, Rory D. de Vries^2^, Virgil A.S.H. Dalm^5,6^

1. Department of Dermatology, Erasmus University Medical Center, Rotterdam, the Netherlands
2. Department of Viroscience, Erasmus University Medical Center, Rotterdam, the Netherlands
3. Laboratory Medical Immunology, Department of Immunology, Erasmus University Medical Center, Rotterdam, the Netherlands
4. Department of Dermatology-Center of Pediatric Dermatology/Center of Rare Skin Diseases, Erasmus University Medical Center-Sophia Children’s Hospital, Rotterdam, the Netherlands
5. Department of Immunology, Erasmus University Medical Center, Rotterdam, the Netherlands
6. Department of Internal Medicine, Division of Allergy & Clinical Immunology, Erasmus University Medical Center, Rotterdam, the Netherlands

**Corresponding author:**

V.A.S.H. Dalm

v.dalm@erasmusmc.nl

Dr. Molewaterplein 40, 3015 GD Rotterdam, The Netherlands

**
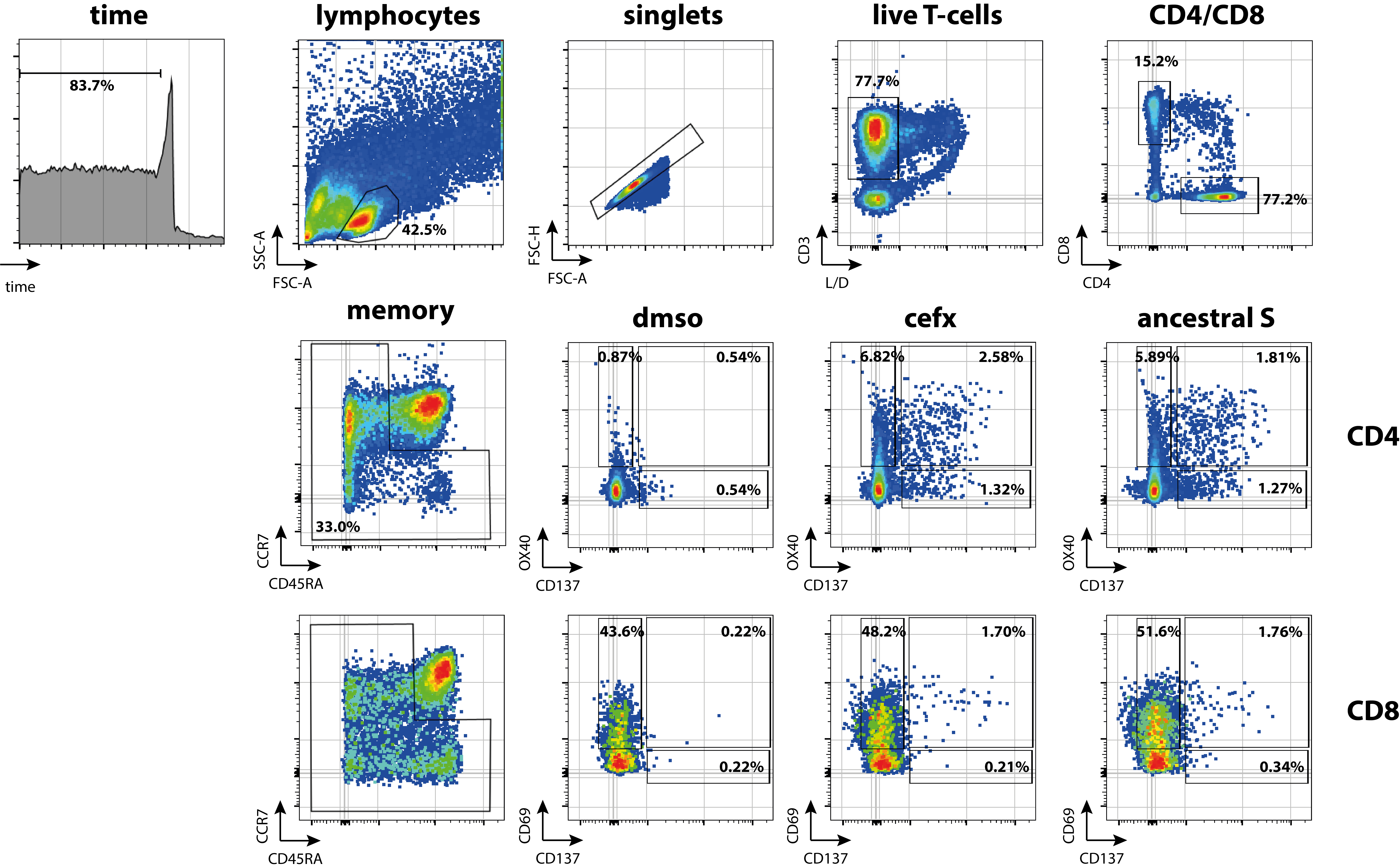
**

**Figure S1. Activation induced marker (AIM) gating strategy.** A time gating was performed to minimalize the contribution of measured artefacts. Next, lymphocytes were gated based on forward (FSC-A) and sideward (SSC-A) scatters. Within the lymphocyte population, singlets were gated by plotting the FSC-A (area) against the FSC-H (height). Next, live CD3+ T-cells were gated and sub-divided into CD4+ or CD8+ T-cells. Within both the CD4+ and CD8+ sub-populations, CCR7+CD45RA+ naïve T-cells were excluded from further analysis. Within the memory T-cell population, activated T-cells were gated as CD137+OX40+ cells in case of CD4+ T-cells and CD137+CD69+ cells in case of CD8+ T-cells. DMSO (negative control), CEFX (positive control), and antigen-specific (ancestral S) AIM gating are shown for CD4+ and CD8+ T-cells for one representative donor.


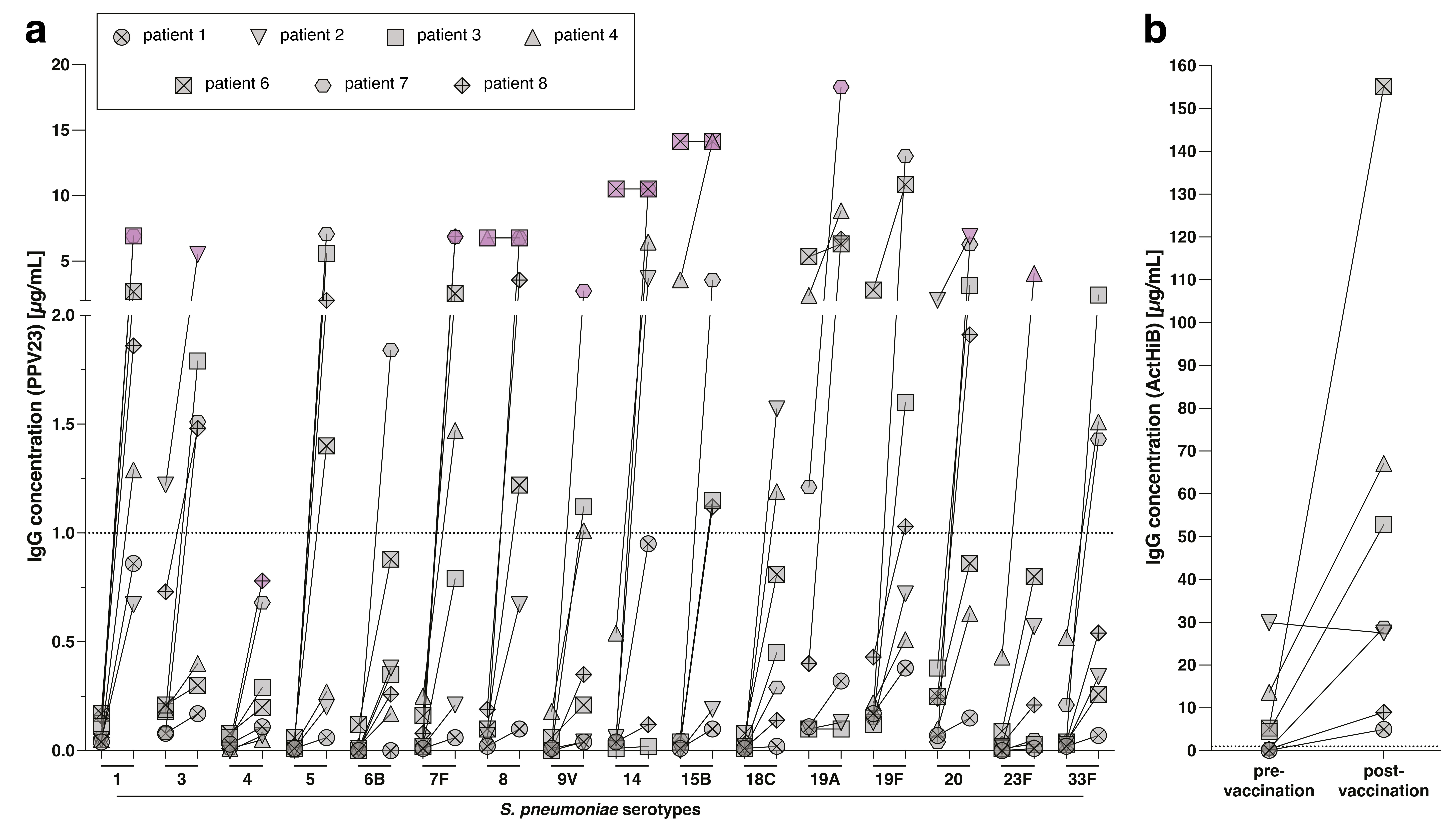


**Figure S2. Vaccination responses of each individual Netherton patients to PPV23 and ActHiB.** (a) Comparison of antibody concentration (µg/mL) per pneumococcal serotype before and after PPV23 vaccination. Samples exceeding the serotype specific upper limit of detection are marked by a purple symbol. (b) Comparison of antibody concentration (µg/mL) before and after ActHiB vaccination. Pre- (left) and post-vaccination responses (right) for each individual Netherton syndrome (NS) patient are connected by lines. Symbols show individual data points. Each NS patient has their own unique symbol as indicated by the legend. Patients 3 and 8 received prior *Haemophilus influenza* type b vaccination, which was part of the Dutch National Immunisation Programme (DNIP) during their childhood. Patient 5 did not receive PPV23 or ActHiB vaccination, antibody concentrations were thus not determined. The dotted line represents 1.00 µg/mL. PPV23= Pneumovax 23, polysaccharide vaccine against *Streptococcus pneumoniae*; ActHiB = conjugate vaccine against *Haemophilus influenza* type b.

**Table S1.** Vaccination responses to PPV23 and ActHiB in NS patients.

| **Vaccine** |  | **Patient 1** | | **Patient 2** | | **Patient 3** | | **Patient 4** | | **Patient 6** | | **Patient 7** | | **Patient 8** | | **Reference value** |
| --- | --- | --- | --- | --- | --- | --- | --- | --- | --- | --- | --- | --- | --- | --- | --- | --- |
| Polysaccharide vaccine  PPV23 |  | Pre^a^ | Post^b^ | Pre | Post | Pre | Post | Pre | Post | Pre | Post | Pre | Post | Pre | Post |  |
| Serotypes | **1** | 0.04 | 0.86 | 0.05 | 0.67 | 0.11 | >6.93 | 0.05 | 1.29 | 0.17 | 2.67 | 0.06 | >6.93 | 0.16 | 1.86 | >1 µg/mL |
|  | **3** | 0.08 | 0.17 | 1.22 | >5.5 | 0.18 | 1.79 | 0.19 | 0.40 | 0.21 | 0.30 | 0.08 | 1.51 | 0.73 | 1.48 | >1 µg/mL |
|  | **4** | 0.03 | 0.11 | 0.00 | 0.07 | 0.06 | 0.29 | 0.01 | 0.05 | 0.08 | 0.20 | 0.01 | 0.68 | 0.03 | >0.78 | >1 µg/mL |
|  | **5** | 0.01 | 0.06 | 0.01 | 0.20 | 0.01 | 5.59 | 0.02 | 0.27 | 0.06 | 1.40 | 0.00 | 7.06 | 0.02 | 2.01 | >1 µg/mL |
|  | **6B** | 0.00 | 0.00 | 0.01 | 0.38 | 0.00 | 0.35 | 0.02 | 0.17 | 0.12 | 0.88 | 0.00 | 1.84 | 0.01 | 0.26 | >1 µg/mL |
|  | **7F** | 0.01 | 0.06 | 0.02 | 0.21 | 0.02 | 0.79 | 0.25 | 1.47 | 0.16 | 2.53 | 0.01 | >6.86 | 0.08 | >6.86 | >1 µg/mL |
|  | **8** | 0.02 | 0.10 | 0.07 | 0.67 | >6.775 | >6.775 | >6.775 | >6.775 | 0.10 | 1.22 | 0.03 | >6.775 | 0.19 | 3.56 | >1 µg/mL |
|  | **9V** | 0.01 | 0.04 | 0.00 | 0.04 | 0.00 | 1.12 | 0.18 | 1.01 | 0.06 | 0.21 | 0.01 | >2.72 | 0.00 | 0.35 | >1 µg/mL |
|  | **14** | 0.04 | 0.95 | 0.06 | 3.66 | 0.01 | 0.02 | 0.54 | 6.46 | >10.51 | >10.51 | 0.04 | >10.51 | 0.04 | 0.12 | >1 µg/mL |
|  | **15B** | 0.01 | 0.10 | 0.00 | 0.19 | 0.04 | 1.15 | 3.57 | >14.143 | >14.143 | >14.143 | 0.05 | 3.54 | 0.03 | 1.12 | >1 µg/mL |
|  | **18C** | 0.01 | 0.02 | 0.02 | 1.57 | 0.01 | 0.45 | 0.03 | 1.19 | 0.08 | 0.81 | 0.01 | 0.29 | 0.01 | 0.14 | >1 µg/mL |
|  | **19A** | 0.11 | 0.32 | 0.10 | 0.13 | 0.10 | 0.10 | 2.38 | 8.84 | 5.34 | 6.32 | 1.21 | >18.29 | 0.40 | 6.69 | >1 µg/mL |
|  | **19F** | 0.17 | 0.38 | 0.13 | 0.72 | 0.12 | 1.60 | 0.22 | 0.51 | 2.80 | 10.85 | 0.15 | 13.02 | 0.43 | 1.03 | >1 µg/mL |
|  | **20** | 0.07 | 0.15 | 2.01 | >6.87 | 0.38 | 3.16 | 0.10 | 0.63 | 0.25 | 0.86 | 0.04 | 6.28 | 0.24 | 1.91 | >1 µg/mL |
|  | **23F** | 0.00 | 0.01 | 0.02 | 0.57 | 0.01 | 0.03 | 0.43 | >4.05 | 0.09 | 0.80 | 0.00 | 0.05 | 0.03 | 0.21 | >1 µg/mL |
|  | **33F** | 0.02 | 0.07 | 0.03 | 0.34 | 0.03 | 2.41 | 0.52 | 1.51 | 0.04 | 0.26 | 0.21 | 1.43 | 0.04 | 0.54 | >1 µg/mL |
| Conjugate vaccine  ActHiB |  | <0.11 | 5.0 | 29.9 | 27.5 | 4.5 | 52.8 | 13.6 | 67.1 | 5.3 | 155.2 | 0.5 | 28.8 | 0.4 | >9.00 | >1 µg/mL |
| Abbreviations: PPV23 = pneumococcal polysaccharide vaccine 23; ActHiB = conjugate vaccine against *Haemophilus influenza* type b. ^a^ = pre-vaccination concentrations; ^b^ = post-vaccination concentrations. | | | | | | | | | | | | | | | | |
